# Strategy to targeting the immune resistance and novel therapy in colorectal cancer

**DOI:** 10.1002/cam4.1386

**Published:** 2018-04-15

**Authors:** Wang Gang, Jun‐Jie Wang, Rui Guan, Sun Yan, Feng Shi, Jia‐Yan Zhang, Zi‐Meng Li, Jing Gao, Xing‐Li Fu

**Affiliations:** ^1^ Department of Pharmaceutics Shanghai Eighth People's Hospital Jiangsu University 200235 Shanghai China; ^2^ Hubei University of Medicine NO. 30 People South Road Shiyan City Hubei Province 442000 China; ^3^ Department of Medicine Jiangsu University Zhenjiang City Jiangsu Province 212001 China

**Keywords:** Colorectal cancer, gene phenotype, immune resistance, immunotherapy, subtypes

## Abstract

Assessing the CRC subtypes that can predict the outcome of colorectal cancer (CRC) in patients with immunogenicity seems to be a promising strategy to develop new drugs that target the antitumoral immune response. In particular, the disinhibition of the antitumoral T‐cell response by immune checkpoint blockade has shown remarkable therapeutic promise for patients with mismatch repair (MMR) deficient CRC. In this review, the authors provide the update of the molecular features and immunogenicity of CRC, discuss the role of possible predictive biomarkers, illustrate the modern immunotherapeutic approaches, and introduce the most relevant ongoing preclinical study and clinical trials such as the use of the combination therapy with immunotherapy. Furthermore, this work is further to understand the complex interactions between the immune surveillance and develop resistance in tumor cells. As expected, if the promise of these developments is fulfilled, it could develop the effective therapeutic strategies and novel combinations to overcome immune resistance and enhance effector responses, which guide clinicians toward a more “personalized” treatment for advanced CRC patients.

## Introduction

Colorectal cancer (CRC) cells may escape immune surveillance and develop resistance to immunotherapy by acquiring genetic alterations. Consequently, some patients exhibit primary or acquired resistance 1. It is generally recognized that tumor–host interaction greatly impacts disease progression and clinical outcome in CRC 2. An interdependence of the tumor mutational landscape and the antitumoral immune response is also highlighted by recent large‐scale cancer genomic projects that have identified a correlation between the expression of immune‐modulatory molecules, cytotoxic T‐lymphocytes (CTLs) infiltration, and the increased mutational load results in the increased production of neoantigens 3.

The previous studies have focused on somatic tumor mutations and tumor immunogenicity, and have known about the expression of gene phenotype that associated regulatory mechanisms on the impact of immune responses in CRC 4, for example, the significant mutational load and high expression of immune checkpoint molecules, which cause substantial immunogenicity in microsatellite instability (MSI) CRC. Recent studies also have identified a stage‐independent survival advantage of CRC patients with abundant CD8 T cells in the tumor microenvironment (TME) 5, 6, 7, 8. Moreover, the disinhibition of the antitumoral T‐cell response by immune checkpoint blockade of the programmed death 1 (PD‐1) pathway has shown remarkable therapeutic promise for patients with mismatch repair (MMR) deficient tumors that characterized by a high frequency of somatic mutations 9.

Among these strategies, assessing the CRC subtypes that can predict the outcome of CRC in patients with immunogenicity, seem to be promising to enhance the immune response for antitumor 10. The aims of this review are to present the available knowledge on the underlying molecular features and immunogenicity of CRC, to discuss the role of possible predictive biomarkers, illustrate the modern immunotherapeutic approaches, and introduce the most relevant ongoing clinical trials. Furthermore, this work is further to understand the complex interactions between the immune surveillance and develop resistance in tumor cells. If the promise of these developments is fulfilled, it could guide clinicians toward a more “personalized” treatment for advanced CRC patients.

## Relationship Between CRC Subtypes and Immune Responses

### Molecular subtype classifications in CRC

Subtype based on gene expression has been developed as a novel technical method for assessing the stratification of disease 11, 12. Some of recent studies have identified the profiling of gene expression in several molecular subtypes of CRC, which was associated with specific clinical outcomes 12, 13. Study has found that the good outcomes for CRC with the lower crypt‐like subtype; however, there was worse survival after relapse that associated with CpGisland methylator phenotype (CIMP) subtype, and worse relapse‐free survival and overall survival were associated with mesenchymal subtype 13.

Furthermore, it has been proved that the elucidated molecular subtypes can predict therapeutic responses for CRC 14. Nevertheless, the effort has discovered that the poor prognosis of CRC was associated with cervical intraepithelial neoplasia (CIN) subtype 15. In addition, the author also demonstrated that a good prognosis and better disease‐free survival (DFS) were related to the hypermutated with frequent BRAF mutations and MSI subtype that characterized by right‐sided preponderance 16, 17. To recognize the molecular biological characteristics of CRC subtypes, investigators further to identify consensus molecular subtypes (CMS) in CRC represent distinct molecular subtypes and genes related to these subcategories of disease as reflected by comprehensive molecular profiling.

**Figure 1:**
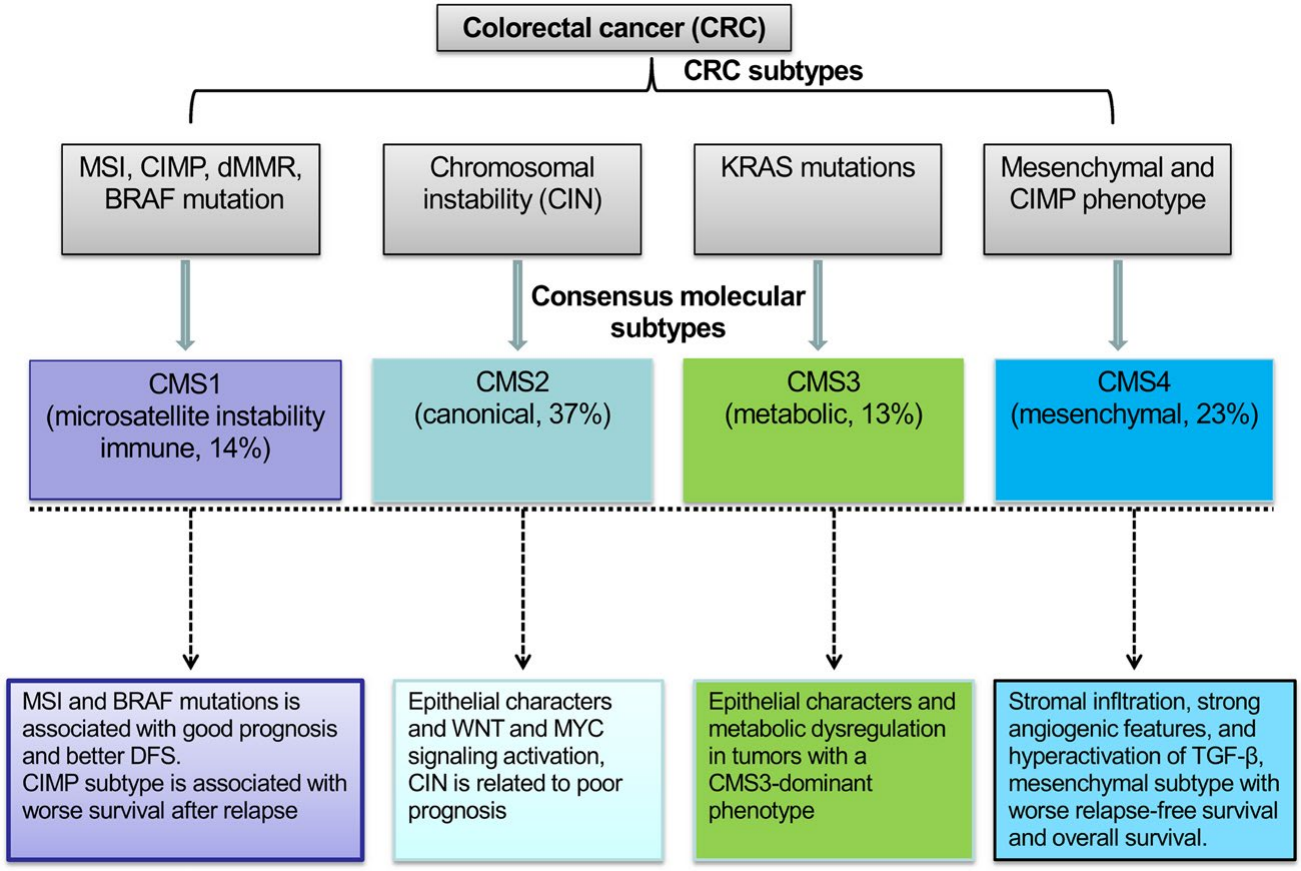
Consensus molecular subtypes (CMS) of CRC subtypes are associated with specific clinical outcomes. CRC is currently classified into four CMS. CMS1 is also called MSI‐like, indicative of hypermutations, and microsatellite unstable features which generally accompany strong immune activation (microsatellite instability immune, 14%). The CMS1 is also enriched for tumors with a CIMP and mutations in the BRAF oncogene; CMS2 (canonical, 37%) is a subtype with high chromosomal instability (CIN), showed epithelial characters and marked WNT and MYC signaling activation; CMS3 (metabolic, 13%) is enriched in tumors with KRAS mutations and shows epithelial characters and metabolic dysregulation, *and KRAS*‐mutated CRC is highly heterogeneous at the gene expression level, with unique metabolic dependencies in tumors with a CMS3‐dominant phenotype; CMS4 (mesenchymal, 23%) has a mesenchymal phenotype and frequent CIMP phenotype and shows stromal infltration, strong angiogenic features, and hyperactivation of transforming growth factors (TGF‐*β*).

Collectively, CRC is currently classified into four CMS and a fifth unclassified group 18. CMS1 is also called MSI‐like, and the features of microsatellite unstable and hypermutations are often accompanied by strong immune activation (microsatellite instability immune, 14%). CMS1 contains most microsatellite unstable tumors with mutations in genes encoding DNA mismatch repair deficiency that often resulting in CRC characterized by a high mutational burden. The CMS1 is also enriched for tumors with a CIMP and mutations in the BRAF oncogene; CMS2 (canonical, 37%) is a subtype with high chromosomal instability (CIN), with the epithelial characteristics and the marked signal activation of WNT and MYC; CMS3 (metabolic, 13%) is enriched in tumors with KRAS mutations with regard to high heterogeneous at the level of gene expression, shows epithelial characters and metabolic dysregulation, and has the unique metabolic dependencies in tumors with a CMS3‐dominant phenotype; CMS4 (mesenchymal, 23%) has a mesenchymal phenotype and frequent CIMP phenotype and shows stromal infltration, powerful angiogenesis, hyperactivate transforming growth factor‐beta (TGF‐*β*) 19. The fifth unclassified groups could be defined as the mixed features (13%) that represented an intratumoral heterogeneity and transition phenotype 20 (Fig. 1).

### CRC subtypes and immunotherapy

To improve CRC patient classification and identify putative molecular targets for immunotherapy, large collections of transcriptomic databases from tumor specimens have been generated 21. Based on the unique global gene expression profiles, several CRC subtypes are now recognized 22. For example, the definition of CMS1 is the combination of upregulated expression in MSI CRC with high immunization penetration and immunization checkpoint. CMS1 is defined as upregulated Th1 lymphocyte, cytotoxic T cell, NK cell infiltration, and upregulated immune checkpoint, such as PD‐1.

**Figure 2:**
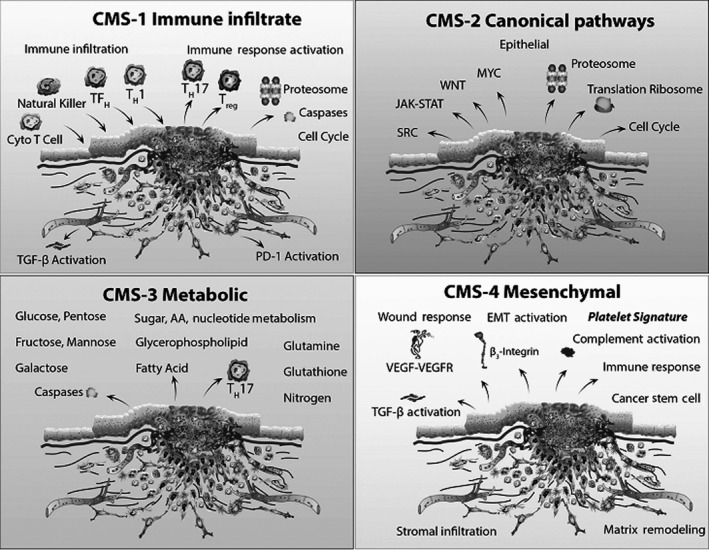
The immune profiles and immune pathways in CRC subtypes. CMS1 is defined by upregulated immune pathways with upregulated Th1 lymphocyte, cytotoxic T cell, NK cell infiltration, and upregulated immune checkpoints such as PD‐1. CMS2 demonstrates upregulation of canonical pathways including WNT and MYC downstream targets. CMS3 is defined by upregulation of metabolic pathways including fatty acid oxidation. CMS4 is the mesenchymal subtype displaying upregulated EMT pathways, TGF‐*β* signaling, matrix remodeling, angiogenesis, complement activation, integrin‐*β*3 upregulation, stromal infiltration, and immune upregulation.

CMS2 shows the upregulation of typical pathways, including the MYC and WNT downstream targets. CMS3 is defined by the upregulation of metabolic pathways such as fatty acid oxidation. CMS4 is the mesenchymal subtype according to adjust the epithelial–mesenchymal transition (EMT) pathway, upregulation, angiogenesis, TGF‐*β* signal matrix reconstruction, and upregulation of integrin‐*β*3 and immune (Fig. 2). Furthermore, a relevant primary example of ecological stratification of colorectal tumor has been established to the infiltration of the tumor mass by subgroups of T cells, which can predict prolonged disease‐free survival following chemotherapy 23, 24.

Despite the immunogenicity of these subtypes, the tumor is known to establish several mechanisms to escape immune surveillance 25. Therefore, different solutions may restore the immune response against these easily targetable cells. To restore patient immunity against cancer cells, diverse strategies may be pursued, including an active immunotherapy (cytokines, immune checkpoint inhibitors, costimulatory pathways, and cancer vaccines) or a passive immunotherapy (adoptive cellular therapy and monoclonal antibodies) approach 6, 26. Of interest, the experts' consensus on the molecular subtype of colorectal adenocarcinomas leads to novel approaches and personalized treatments.

Notably, MSI‐high tumors have a mutational rate 20 times higher than microsatellite stability (MSS) tumors, reflecting the tendency to express a higher load of neoantigens, thus improving the response to immunotherapy 27. CMS1 also includes tumors with methylated CpG islands (CpG island methylator phenotype, CIMP‐H), which often results in gene silencing, and tumors with mutations in the BRAF oncogene 27, 28. Interestingly, this subgroup displays a diffuse immune infiltrate; moreover, CMS1 subtype exhibits high expression of T cell‐recruiting chemokines as well as the expression of Th1 cytokines that have been shown to correlate with good prognosis in CRC 29. Indeed, DNA mismatch repair deficiency (dMMR) causes a high mutational oncogenic load, such as frameshift mutations and neoantigen expression, which can induce an active immune microenvironment reaction characterized by a high density of TILs 30. Further investigations have explored the association between neoantigens and immune infiltrate in CRC. A higher neoantigen load is shown to be associated with a high lymphocyte score and with increased CRC‐specific survival 31. Therefore, tumors with a high neoantigen load would seem to benefit more from immunotherapy 27.

### The biological link between immune profiles and CRC subtypes

Galon and colleagues first demonstrated the relevance of specific immune signatures in the prognosis of early‐stage CRC 6, 32, 33. High lymphocyte infiltration in primary CRC tumors, particularly CTLs and TH1 cells with an *γ*‐interferon (IFN‐*γ*)‐dominant immune profile, positively correlated with relapse‐free and overall survival 32, 33. Conversely, TH17 cell infiltration and an interleukin‐17 (IL‐17)‐dominant immune profile was associated with poor outcomes. In addition to the highly immunogenic hypermutated MSI subtype of CRC, the expression‐profile analysis identified another cluster of tumors that displayed a different immune infiltration pattern, which showed high expression of genes specific to Treg cells, myeloid‐derived suppressor cells (MDSCs), monocyte‐derived cells and TH17 cells, which were typically seen in the microenvironment of immune‐tolerant malignancies 34.

A clinical translation of these findings was to establish a scoring system, called “immunoscore,” which based on two distinct types of lymphocyte populations (CD8+ CTLs and CD45RO memory T cells) at the tumor center and at its invasive margin 6. Their quantification in early‐stage CRCs was a validated prognostic marker, with 50% less risk of tumor relapse for those tumors with high immunoscores versus those with low immunoscores 35. Subsequently, investigators have shown that the density of T cells decreased along with tumor progression, whereas the densities of B cells and T follicular helper (TFH) cells increased from early‐stage to more invasive CRC 36. High B‐cell or TFH infiltration in late‐stage neoplasms correlated with prolonged disease‐free survival 32. Moreover, immune infiltration patterns and inflammatory cytokines have been linked to microbial dysbiosis and colon carcinogenesis. The tumor infiltrates CD4+ T cells that expressed the transcription factor of FOXP3 (FOXP3), which acts on the regulatory T (Treg) cells that impeded the effective immune response to cancer cells, show significantly worse prognosis 37.

The biological link between the inflamed immune CRC subtype is characterized by marked upregulation of immunosuppressive factors, such as TGF‐*β* and CXCL12, and high expression of genes encoding chemokines that attract myeloid cells, including chemokine (C–C motif) ligand 2 (CCL2) and the related cytokines IL‐23 and IL‐17, which are known carcino‐genic drivers in colitis‐associated CRC 38. Recent work also indicates that the stroma of CMS4 tumors is infiltrated not only with endothelial cells and CAFs but also with innate immune cells 39. In addition, it suggests that the worse outcomes seen in the CMS4 mesenchymal population may be partially linked to the pro‐metastatic inflammatory microenvironment. These results corroborated initial findings by Galon and others that an activated immune microenvironment in early‐stage CRC was a strong determinant of the risk of distant dissemination and was associated with an aggressive clinical behavior 40.

Taken together, these findings suggest that the molecular CRC subtypes might be associated with specific clinical outcomes and the relevance of specific immune signatures in the prognosis of early‐stage CRC, molecular subtype of colorectal cancer may lead to novel approaches and personalized treatments. The biological link between the inflamed immune CRC subtype is characterized by marked upregulation of immunosuppressive factors which may be a promising chemopreventive and/or chemotherapeutic strategy against CRC (Fig. 2). However, more molecular and genetic approaches are required to understand the exact molecular subtype of CRC and immune profiles and pathways in regulation of immune responses against CRC cells.

## Strategies to Therapy Colorectal Cancer by CMS Subtypes

### Targeting therapy for CMS1, 2, 4 subtypes in RAS wild‐type CRC

In CMS1 subtypes of CRC, there are some studies that showed the reduced expression of the EGFR ligands amphiregulin (AREG) and epiregulin (EREG), and this reduced expression is linked to hypermethylation of the ligands' promoter regions 41. It is also known that distal carcinomas, particularly of CMS2 phenotype, frequently overexpress EGFR ligands and harbor amplifications of EGFR and insulin receptor substrate 2 (IRS‐2) 41, 42, which are the markers of cetuximab sensitivity 43. But additional oncogene alterations that potentially drive resistance to EGFR mAbs in RAS wild‐type patients are also enriched in the CMS2 population, including actionable HER2/neu (also known as ERBB2) and insulin‐like growth factors 2 (IGF2) copy number gains, making it the most appealing group to test combinations of pan‐ERBB and IGF1R inhibitors 44.

On the contrary, RAS wild‐type tumor with a mesenchymal phenotype seems to be intrinsically resistant to anti‐EGFR agents in preclinical models. In fact, retrospective biomarker analyses of a patient cohort in the chemotherapy‐refractory setting and a randomized clinical trial in the chemonaive setting suggest no benefit of treatment with cetuximab in patients with mesenchymal‐like tumors 45. The major goal to identify the actionable targets in CMS4 phenotype is considering the higher chances of metastatic spread 46. There is strong evidence that stromal cells mediate resistance of CRC cell lines to chemotherapies and targeted agents 47. Indeed, the retrospective analysis of a randomized clinical study shows that the tumor with mesenchymal phenotypes of patients, and there is a poor prognosis and no benefit from adjuvant chemotherapy of oxaliplatin in phase III of patients with CRC 48.

**Figure 3:**
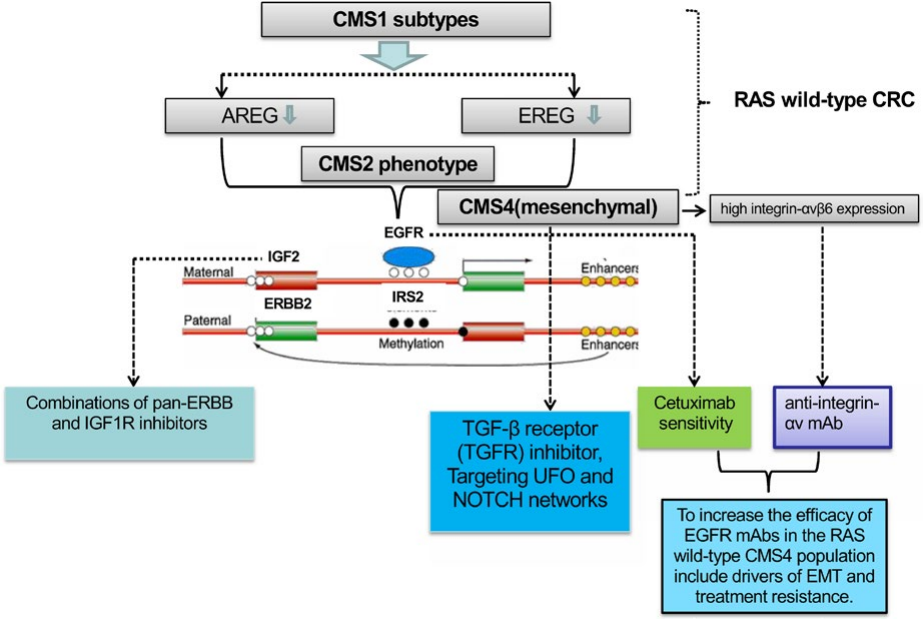
Targeting therapy for CMS1,2,4 phenotype in RAS wild‐type CRC. In CMS1 subtypes of CRC, the reduced expression of the EGFR ligands amphiregulin (AREG) and epiregulin (EREG) is linked to hypermethylation of the ligands' promoter regions. In CMS2 phenotype, frequently overexpress EGFR ligands and harbor amplifications of EGFR and IRS2, which are markers of cetuximab sensitivity. However, the resistance to EGFR mAbs in RAS wild‐type patients is also enriched in the CMS2 population, making it the most appealing group to test combinations of pan‐ERBB and IGF1R inhibitors. Indeed, both UFO and NOTCH networks pathways are overactive in CMS4 mesenchymal CRC. The combination of chemotherapy with a TGF‐*β* receptor (TGFR) inhibitor has tested positive for a “TGF‐*β* activated” signature as part of project in metastatic CRC. The effort to discover the potential targets that may increase the efficacy of EGFR mAbs in the RAS wild‐type CMS4 population includes drivers of EMT and treatment resistance, such as MET and integrins, and combination therapy with cetuximab and a mAb anti‐integrin‐*α*v was particularly effective in patients whose tumors displayed high integrin‐*α*v*β*6 expression levels in CMS4 mesenchymal samples.

Notably, the use of TGF‐*β* signaling inhibitors to block the crosstalk between cancer cells and the microenvironment was shown to halt disease progression of stromal‐enriched poor prognosis CRC tumors 49. Furthermore, the combination of chemotherapy with a TGF‐*β* receptor (TGFR) inhibitor has already moved to clinical trials in patients whose tumors test positive for a “TGF‐*β* activated” signature as part of project in metastatic CRC 50. Similarly, signaling activation of UFO (a tyrosine‐protein kinase receptor encoded by AXL) and NOTCH network also triggers EMT in CRC and is associated with an aggressive tumor phenotype and resistance to targeted agents 51. Indeed, both pathways are overactive in CMS4 mesenchymal CRC, thereby providing novel leads for pharmacological inhibition in this metastasis‐prone subtype of the disease (Fig. 3).

Nevertheless, the effort to discover the potential targets that may increase the efficacy of EGFR mAbs in the RAS wild‐type CMS4 population includes the drivers of EMT and treatment resistance, such as mesenchymal–epithelial transition (MET) and integrins 52. In a clinical report, combination therapy with cetuximab and a mAb anti‐integrin‐*α*v was particularly effective in patients whose tumors displayed high integrin‐*α*v*β*6 expression levels 53, which was typically seen in CMS4 mesenchymal samples 54.

### The potential targets for metabolic CMS3 phenotype in CRC

Both at gene and protein level heterogeneously expressed within KRAS‐mutated colorectal tumors, with unique metabolic dependencies in tumors with a CMS3‐dominant phenotype 55. Studies further found that samples were classified as CMS3 (is characterized by a general dysregulation of metabolism), and 13% of CMS3 cases were characterized by high KRAS mutation rates 56. Although therapy‐optimization strategies in patients with RAS wild‐type CRC are unlimited, targeted treatment of KRAS‐mutated disease has proved extremely difficult and has not evolved in recent years 57. For instance, despite strong scientific rationale and preclinical data supporting the combination of MEK inhibitors and PI3K pathway inhibitors, no clinical activity was seen in CRC 58.

Recent studies have shown a strong causal association between KRAS mutations and ^18^F‐FDG accumulation in patients with metastatic CRC 59. Context‐specific molecular susceptibilities have been identified in KRAS‐mutated tumors, such as deficits in nucleotide metabolism and lysosomal maturation 60. Interestingly, the subgroup of KRAS‐mutated tumor with coexisting amplifications of the KRAS‐mutated allele is characterized by marked rewiring of glucose metabolism toward glutathione biosynthesis, mirroring the CMS3 metabolic adaptation seen in CRC 61. Importantly, KRAS and BRAF may contribute to colorectal cancer phenotypes via metabolic reprogramming; for example, the DLD‐1 and RKO colorectal cancer cell lines, which have oncogenic mutations in KRAS and BRAF, display increased expression of the primary glucose transporter SLC2A1 (commonly known as GLUT‐1) and exhibit a Warburg effect phenotype, with the increased glucose consumption rate and concomitant increased lactate production rate in isogenic colorectal cancer cells 62.

**Figure 4:**
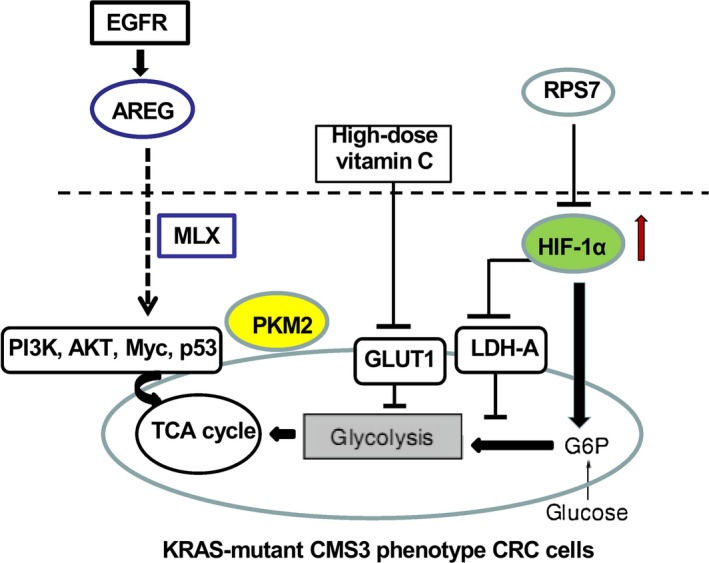
The potential targets for mutated KRAS metabolic CMS3 phenotype in CRC. KRAS and BRAF may contribute to colorectal cancer, display increased expression of the primary glucose transporter SLC2A1 (GLUT‐1), and exhibit a Warburg effect phenotype, with the increased glucose consumption rate and concomitant increased lactate production rate in isogenic colorectal cancer cells. Therefore, the global energy metabolism of CRC cells could be controlled by KRAS mutations to promote glucose uptake, while the control toward Warburg effect is critical to connect tumor cells with complex genetic changes, such as PI3K, AKT, Myc, HIF‐1, p53. The role of LDH‐A in the invasive colorectal cancers to maintain an efficient glycolysis in glycolytic phenotype and the LDH level correlates with the change in overall tumor burden in CRC. Remarkably, high‐dose vitamin C has been shown to impair CRC tumor growth in mouse models by causing oxidative stress mediated the increased uptake of the oxidized vitamin C through GLUT‐1 and this inhibited glycolysis at glyceraldehyde‐3‐phosphate dehydrogenase (GAPDH) to result in an energy crisis and ultimately cell death.

In the GLUT‐1 mRNA overexpression with KRAS mutation‐positive tumors, the KRAS oncogene is upregulated in the first steps in the pathway of glucose metabolism. Therefore, the global energy metabolism of cancer cells could be controlled by KRAS mutations to promote glucose uptake, while the control toward Warburg effect is critical to connect tumor cells with complex genetic changes, such as PI3K, AKT, Myc, HIF‐1, p53 63, 64. Noteworthily, the high glycolytic profile is an essential metabolic pathway in the hypoxia for KRAS‐mutated patient survival, when treated them with Bevacizumab plus chemotherapy 65. Therefore, the plausible hypothesis is that the anti‐angiogenic factors of cloned cancer to develop the early and/or late resistance may be more suitable for redundant and rapid metabolic changes or glycolysis in anaerobic environments induced by therapy 66 (Fig. 4).

Moreover, the increased expression of PKM2 protein is closely related to serum CEA level and TNM stage, and the prognosis of patients with CRC is poor. At the same time, PKM2 protein expression is the independent prognostic factor of the overall survival (OS) of patients with CRC 67. Furthermore, the glycolytic enzyme, LDH‐A, is encoded by the LDH‐A gene and plays key role in glucose metabolism, which is a critical branch point in cancer cells. In CRC cells, pyruvate is either reduced to lactate by LDH‐A, instead of the former undergoing oxidative decarboxylation to produce Acetyl CoA for early steps in glycolysis 68. Metabolically, the conversion of L‐lactate and NAD to pyruvate is catalyzed by LDH‐A with nicotinamide adenine dinucleotide phosphate (NADPH) and NAD^+^ conversion. Compared with normal mucosa, it has been found that liver metastatic tissues have the higher levels of LDH‐A expression than in the primary colorectal cancers, with its remarkably upregulated LDH‐A levels 69. Indeed, the glucose uptake, production of lactate, and intracellular ATP levels are decreased by LDH‐A knockdown in CRC cells 69.

These findings suggested the critical role of LDH‐A in the invasive colorectal cancers to maintain an efficient glycolysis in glycolytic phenotype. The changes in LDH level were correlated with the overall burden of tumor in CRC. At the same time, the level of LDH was observed to be unusually high in the detection of pseudoprogression. Clinical study has reported on the effect of LDH on the pseudo and real progress in immune‐targeting therapy.

Remarkably, high‐dose vitamin C has been shown to impair CRC tumor growth in mouse models by causing oxidative stress mediated the increased uptake of the oxidized vitamin C through GLUT‐1 and this inhibited glycolysis at glyceraldehyde‐3‐phosphate dehydrogenase (GAPDH) to result in an energy crisis and ultimately cell death 70. Novel inhibitors of metabolic enzymes, such as glutaminase and fatty acid synthase (FASN) 71, 72, are in early clinical development and should be revisited as targeted interventions in KRAS‐mutated CRC.

### Toll‐like receptor (TLR) 3 in KRAS‐mutated CRC

Toll‐like receptor (TLR) 3, the family member of the toll‐like receptor family of the host innate immune system, is the pattern recognition theme of dsRNA pathogens 73. Over the past decade, the focus has shifted from the treatment of patient experience to the precise approach that is dominated by biomarkers. The discovery of cancer, especially in the field of molecular pathology, has formed a personalized medical model 74. Under this background, the TLR3 mutation can be used as KRAS‐mutant CRC patients with good therapeutic targets, and in the context of KRAS mutations, environment is more effective, and the cut will further enhance its efficacy of TLR3, thus more particularly effective in KRAS mutations in patients with the queue 75.

There are several TLR agonists that are rarely approved by the FDA as an adjuvant to immune stimulation for patients with cancer 76. Instead, there are not many TLR antagonists and no toxicity or safety. TLR 3, 4, and 9 antagonists are being developed for research purposes, but are far from ready for therapeutic trials. Although there is no good TLR3 antagonist, some small molecules are testing the TLR3 downregulation efficacy 77. Another strategy for TLRs reduction can be achieved by developing and managing neutralizing antibodies against specific TLRs 78. This area requires a large amount of research, combined with molecular strategies, to improve the therapeutic ability of tumor viruses.

The commercial HEK‐BlueTM‐hTLR3 cell is expressed through TLR3, which is the subject of host pattern recognition and is responsible for the detection of the virus. In addition, the investigation also showed that the effective expression of the host TLR3 was established by establishing a robust innate immune response with the KRAS‐mutant HCT116 cell line 79, which inhibited the potential of the virus infection. By lowering the expression of TLR3 and siRNA, the anticancer activity of the virus was improved. In vivo experiments, study further confirmed the role of TLR3 in inhibiting the virus by increasing the tumor response rate in athymic mice that xenotransplanted human CRC cells. Strategies to mitigate TLR3 responses can be used as a tool to improve the efficacy of the retrovirus, specifically targeting the transmission of KRAS‐mutated CRC 80.

These findings clearly show that the inhibition of TLR3 expression inhibits the KRAS‐mutant cancer cells into a better therapeutic target for the oncolytic reovirus. This study needs to describe TLR3 at the molecular level in the process of re virus infection and reproduction mechanism, and special emphasis on the condition of KRAS‐mutant CRC, in search of a better method for the treatment of CRC with KRAS mutations.

### Prospect of drug development for CRC subtypes

Whether the relevant features of CRC subclassification, stromal and immune cells may still predict the responses of differential drug remains unknown. This may be because the drugs themselves, which can have a promiscuous mechanism of action, may not be tracking and single way descriptors, or we will not be able to correctly define the involved pathways or crosstalk using static omics data 81. Furthermore, the insights into drug matches for specific gene expression or immune CRC subtypes discussed here are based on preclinical hypotheses or retrospective exploratory analyses of clinical cohorts with associated shortcomings. The underlying understanding mechanism for the treatment of sensitivity or resistance requires a robust development of the biomarker discovery process using the system biological approach and the data set of orthogonal interrogations 82, 83. In addition, any emerging biomarker has to be put into context with driver gene mutations, MSI status, CMS, and immune CRC classifications 17, 84.

These above results laid the foundation for future research, and the study assessed the presumption of tumor‐specific antigen expression of related genes and regards it as the prognosis of metastatic tumor biomarkers, which especially classified as “mesenchymal” or “metabolic” tumors. In addition, studies have shown that personalized immunotherapy can provide treatment opportunities for patients with MSS CRC tumors. With regard to CMS classification in a research setting, the available models need to be optimized for subtype prediction on tissues in which microenvironment content is different from primary colorectal tumors, such as metastatic lesions and PDXs 85.

Overall, advances in patient stratification and drug development strategies have to be rapidly translated from the metastatic to the adjuvant setting 86, 87. Most researchers agree that a better understanding of the drivers of this pro‐metastatic state will guide drug selection in future biomarker‐driven adjuvant clinical trials, hopefully, for improving the survival of CRC.

## The Mechanisms of Acquiring Immune Resistance in CRC

### Immune resistance in CRC

Immunotherapy has led to clinical benefits in some cancer patients' therapy. However, in cancer treatment, the great challenge is developing disease progression and/or drug‐resistant diseases after treatment. The first kind of resistance is a special form of Darwinian natural selection that comes from before treatment intervention in the tumor mass pre‐existing genetic or epigenetic traits selected 88. The main driver of the immune‐resistant tumor cell mutation that is produced by this mechanism seems to be the genome and later instability of the transformed cell. Darwinian selection of antidrug cloning from the tumor cell population could lead to the development of tumor cell mutations and the gene and epigenetic characteristics that the ability to evade the treatment. The second resistance to immunotherapy is achieved at the level of individual tumor cells 89. This is because the cancer cells can alter their gene expression to respond to immune cells or their products. This form of acquired resistance may use adaptive mechanisms and immune homeostasis, which also called as homeostatic resistance.

An obvious example of this resistance is the PD‐L1 expression in response to the IFN‐*γ* secretion when induced cancer cells, so the T cell destroys the tumor cells in vivo model 90. It has already cleared that some patients that initially responded to the treatment of the anti‐PD‐L1 treatment, even though they were still being treated. Possible reasons as follows: insufficient penetration CD8+ T cells, the reaction of monoclonality, loss of neoantigens, the lack of IFN signal, excessive loss of PD‐1 infiltrating T‐cell receptors, or other immune upregulation checkpoint. The general mechanism of treatment is that the acquired resistance may be very similar to naturally acquired resistance.

The long‐term clinical benefits of the mutation‐derived neoantigens may be predicted by anti‐CTLA4 (ipilimumab) and the anti‐PD‐L1 (pembrolizumab) treatment 80. The neoantigen is defined in a patient's tumor, the neoantigen‐specific T‐cell response tumor regression, suggesting the association between the T‐cell response and the antitumor effect of the anti‐PD‐L1 treatment. Of course, the natural immune function of the human tumor is not easily studied in the absence of genetic tools and controls provided by animal research. But now a new generation of sequencing technology progress and specific to the individual in antigen epitope prediction allows people to define the T‐cell response in individual patients 91 and should allow natural immune with a history of the patient's tumor to be followed before and after treatment.

One of the early examples of such a high degree of microsatellite instability (MSI—high) in CRC is associated with intense T‐cell infiltration, because of the MSI‐high tumor frameshift mutation and truncated protein (neopeptides) caused by mismatch repair defects, the antitumor T‐cell‐mediated adaptive immunity 92, 93. However, MSI status is not the only determinant of the immune response to colorectal cancer, because the number of tumor infiltrating T cells overlapped significantly with MSI‐high and microsatellite stability (MSS) in CRC 94. Interestingly, clinical trials that assessed PD‐1 inhibitor immunotherapy in patients with CRC have recruited only small cohorts of patients with mCRC 95. Studies on the tumor microenvironment are based on archival specimens with different antibody PD‐1 and PD‐L1 preparations for immunohistochemistry, independent from immunotherapy trials. Immunotherapy with PD‐1 therapy has potential benefit for immunogenic MSI‐H CRCs, whereas there is no evidence to date to suggest immunotherapy benefit in MSS CRCs 96, 97.

**Figure 5:**
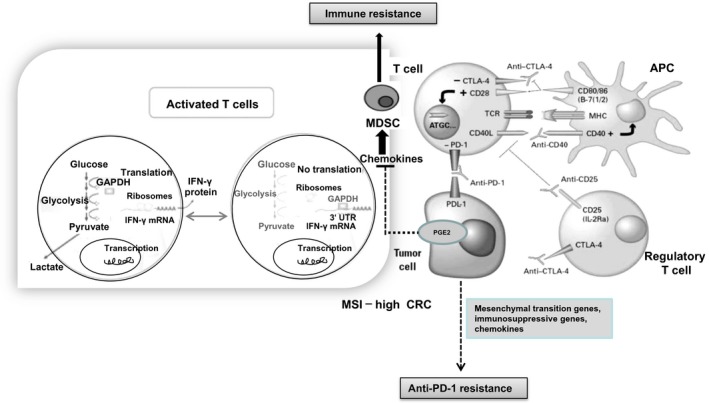
The immune resistance and immunotherapy in MSI‐high CRC. The high degree of microsatellite instability (MSI—high) in CRC is associated with intense T‐cell infiltration, caused by mismatch repair defects in MSI‐high tumor frameshift mutations and truncated protein (neopeptides), causing antitumor T‐cell‐mediated adaptive immunity. Immunotherapy with PD‐1 therapy has potential benefit for immunogenic MSI‐H CRCs whereas there is no evidence to date to suggest immunotherapy benefit in MSS CRCs. Tumor cells may escape immune surveillance by acquiring different genetic alterations, a higher expression of mesenchymal transition genes, immunosuppressive genes, and chemokines that recruit immunosuppressive cells may be associated with innate anti‐PD‐1 resistance. Likewise, high tumor PGE2 expression represents a key mediator of immune resistance, mainly due to the secretion of suppressive chemokines and the recruitment of MDSCs, which results in immunogenic loss.

It appears that the tumor cells may escape immune surveillance by acquiring different genetic alterations. Indeed, some patients exhibit an innate resistance to immunotherapy. A higher expression of mesenchymal transition genes (AXL, FAP, LOXL2, ROR2, TWIST2, TAGLN, and WNT5A), immunosuppressive genes (VEGFA, VEGFC, and IL‐10), and chemokines that recruit immunosuppressive cells (CCL2, CCL7, CCL8, and CCL13) may be related to innate anti‐PD‐1 resistance 98. By contrast, some patients quickly develop resistance, even after an initial benefit with a significant reduction in tumor burden, suggesting that a rapidly proliferating resistant clone may cause the progression of resistance 99, 100. As a result, these mutations cause decreased antigen presentation and immune escape 101 (Fig. 5). Likewise, high tumor PGE2 expression represents a key mediator of immune resistance, mainly due to the secretion of suppressive chemokines and the recruitment of MDSCs, which results in immunogenic loss 102.

Because anti‐PD‐L1 treatment improved significantly, however, the immune therapy faced many problems 103, 104, including those traditional treatment that induced therapeutic resistance, as well as immune‐related adverse events. The biggest challenge for immunotherapy is rationalizing, but the expansion of its utility.

### EMT and immune resistance

Epithelial interstitial transition (EMT) is the basic process of distant metastasis of cancer. During EMT, epithelial cells expressed mesenchymal genes that transformed their phenotypes from the epithelium to mesenchymes, allowing the cells to invade the tumor matrix and blood vessels. Studies have shown that the excessive expression of snail not only accelerated the invasion in cancer cells, but also produced to induce immune regulation of dendritic cells (regDC) induced immunosuppression. 105, 106.

The diversity of immunocheckpoint inhibitors for patients with colorectal cancer may be due to heterogeneity and heterogeneity in tumors 107. Although heterogeneous components interact with each other, it is necessary to clarify the immunological significance of each component to reveal potential mechanisms for further immunotherapy. Using large databases such as TCGA and recent comprehensive analysis, it is expected that more axes will be highlighted to assess the patient's heterogeneity 108, 109.

### Tumor‐infiltrating lymphocytes (TILs) and CCL5

Immunological scoring, classification of the type, location, and number of tumor infiltrating lymphocytes (TILs), has been revealed as one of the strongest prognostic markers of CRC 110, 111. The immunological score quantifies the density of cytotoxicity (CD8 +) and memory T cells (CD8 +/CD45RO+ or CD3 +/CD8 +, CD3/CD45RO) at the center and aggressive edge of CRC 7, 23. The higher immunological score indicates that the infiltration of CD8+ and CD45RO+ T lymphocytes increased the central tumor and invasive margin, which is a positive prognostic marker 23.

Interestingly, the number of TILs and MSI status do not fully predict the prognosis of patients with CRC. One set of CRCs is described as having a characteristic of high lymphogenetic expression, and somewhat unexpected and is associated with poor prognosis 57. These CRCs also add myeloid infiltration and mesenchymal. Recently, a group of patients with melanoma also had a mesenchymal signature, which was considered resistant to checkpoint blockade 112. The signature is composed of genes associated with EMT, immunosuppressive genes, monocytes/macrophages.

Studies have identified a unique microenvironment in immunology of CRC hepatic metastasis in the context of increased cytokines and chemokines in macrophages 113. For example, the elevated CCL5 levels, which are secreted by T cells, result in tumor cell proliferation, invasion and increase in the production of matrix metalloproteinase, which is associated macrophages with tumor cells 114, 115. Improved treatment strategies for patients with CRC are clearly needed, and immunotherapy is very promising. Unfortunately, MSS CRCs have a large degree of resistance to immunotherapy, such as a single agent of checkpoint blockade 116. However, the tumor microenvironment is related to this in a number of studies [117]; however, this is a very small study, and it does not achieve its main endpoint of the curative effect for CCR5 inhibition.

## Strategy to Overcome the Acquire Immune Resistance in CRC

### Regulation of the antitumor T‐cell immunity

Resistance to chemotherapy and immune‐suppressive milieu around the tumor cells remain major obstacles in effective anticancer treatments 118, 119. Numerous evidences indicate that cancer cells cause suppressive effects on the host immune system, particularly cell‐mediated immune response resulting in relapse and progression of tumors 120, 121. Regulatory T cells (Tregs) are a heterogeneous subpopulation of T lymphocytes, which play a crucial role in tolerance maintaining 122. Tregs are CD4+ lymphocytes characterized by constitutive expression of CD25 and Foxp3 that is the key regulatory gene for the function and development of CD4+ CD25+ Treg 123. Tregs inhibit the local immune response, decreasing T‐lymphocyte proliferation and pro‐inflammatory cytokine secretion which promote tumor progression 124, 125. Also, some studies showed that Tregs are resistant to conventional chemotherapy which may help tumor cells from immune evasion 126.

**Figure 6:**
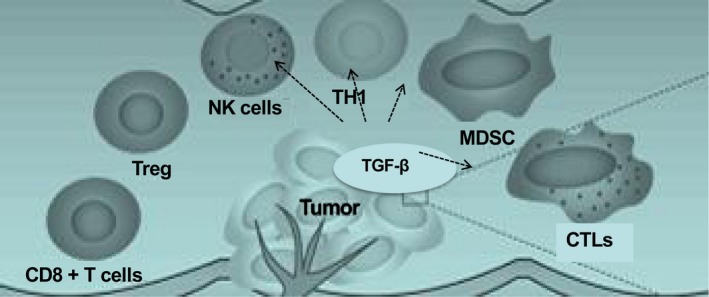
Regulation of the antitumor T‐cell immunity‐mediated TGF‐*β* in CRC. The pro‐tumorigenic functions of TGF‐*β* are mediated not only through direct action on tumor cells but also through its effects on immune cells—inhibition of CTLs, TH1 cells, and NK cells, and expansion of Treg cells, B cells, and MDSCs. CTLs can be found in the tumor core or in the tumor margin. The positive treatment outcome was associated with an expansion of tumor‐infiltrating effector CTLs and TH1 cells, enhanced antitumor T‐cell immunity. Alternative immunotherapeutic approaches to be explored in inflamed TGF‐*β*‐mediated mesenchymal tumors include pharmacological elimination of MDSCs or blockade of related immunosuppressive chemokine signaling circuits and pathways in an immune‐evasive microenvironment.

The idea is to overcome multiple mechanisms that mediate immune tolerance to self‐antigens and block the intense immunosuppressive response in the tumor microenvironment 127. It is not only tumor cells direct act on the pro‐tumorigenic TGF‐*β* functions but also immune cells are mediated by its effects, such as inhibition of CTLs, TH1 cells, and NK cells, and expansion of Treg cells, B cells, and MDSCs 128, 129. Therefore, for an immunotherapy to be successful in inflamed mesenchymal tumors, it is likely to require not only inhibitors of T‐cell suppression but also an agonist to boost function of effector CTLs 130, 131 (Fig. 6).

CTLs can be found in tumor edge or tumor core 132. However, lymphocyte infiltration into the tumor is not a stable process. The proportion of infiltrating lymphocytes in tumor tissues increased or decreased significantly during the course of treatment even during the use of immunoregulatory drug for therapy 131. In mouse models of highly aggressive mesenchymal CRC tumors, a potential synergistic effect was observed with the combination of a TGFR inhibitor with a PD‐1 checkpoint inhibitor 133, or with an agonistic OX40 mAb 134, which enhanced effector function and survival of activated T cells. The positive treatment outcome was associated with an expansion of tumor‐infiltrating effector CTLs and TH1 cells, enhanced antitumor T‐cell immunity 135, and a high tumor‐specific IFN‐*γ* response 136. Alternative immunotherapeutic approaches to be explored in inflamed TGF‐*β*‐mediated mesenchymal tumors include pharmacological elimination of MDSCs or blockade of related immunosuppressive chemokine signaling circuits and pathways, as demonstrated in other malignancies with an immune‐evasive microenvironment 38.

### Novel combined approaches to enhance immunotherapy

#### Combination of small molecules and checkpoint inhibitors

For poorly immunogenic or immune‐ignorant CRC tumors, complementary therapeutic approaches to checkpoint inhibitors are also needed 137. These include cancer vaccines with dendritic cells to stimulate tumor infiltration with antigen‐specific CTLs 138, or alternative agents that can enhance T‐cell infiltration and increase expression of T‐cell chemokines in a nonantigen‐specific way, such as histone deacetylase (HDAC) inhibitors 139. Despite negative results with checkpoint inhibitors as monotherapies in patients with tumors that show MSS, multiple trials are investigating the value of combined administration of standard chemotherapies known to induce immunogenic death of CRC cells, such as oxaliplatin 140, and anti‐angiogenic agents that may neutralize vascular barriers preventing T‐cell homing in the microenvironment, including bevacizumab 141. Importantly, it is still unclear to what extent chemotherapies and targeted agents affect the tumor microenvironment (TME).

More specifically, immunotherapy alone appears to have modest success, likely due to the complexity of the TME. Therefore, recent trials have been evaluating novel combined approaches, such as immune‐chemotherapy or combo immunotherapy, that could be more effective than chemotherapy or immunotherapy alone 142, 143. Moreover, some authors observed that VEGF‐A block may help to make the T cells sensitize to the treatment of anti‐PD‐1, and high level of VEGF‐A may be involved in the resistance for treatment of CRC mouse model 144. Interestingly, the anti‐VEGFA antitumor effect is at least partly due to CD8+ T cells, as the CD8+ T‐cell depletion reduces the antitumor effect during the treatment of anti‐VEGFA. Therefore, these data suggest a potential rationale for the association between anti‐angiogenic molecules and checkpoint inhibitors, with particular interest for VEGF‐A‐producing tumors.

It has been observed that MEK inhibition induced the accumulation of T cells within the tumor cells and the major histocompatibility complex (MHC) class I upregulation in mouse models, and controlled the synergy and immune checkpoints, promoted sustainable tumor regression 145. In fact, the preliminary data of the clinical trial evaluated the combination of anti‐PD‐1 and MEK inhibitor, showing early indications of the efficacy of MSS non‐hypermutated CRC patients 146. Another strategy under investigation is the combination of immune modulators and anti‐EGFR therapy in a RAS wild‐type population, reflecting the notion that the immune system substantially contributes to the therapeutic effects of mAbs 147, 148.

It will be interesting to verify whether combining immunotherapy with chemotherapy and/or biological therapies (anti‐EGFR or anti‐VEGF) could produce a synergistic effect in CRC. Obviously, many clinical trials would require to evaluate the efficacy and safety of these novel approaches.

#### Combination of COX inhibitors and checkpoint inhibitors

It would also be useful to understand how to enhance immunotherapy, increasing the effector response and reducing the inflammatory component. Indeed, tumor cells can exploit inflammation for cancer promotion. COX‐2 deregulation plays a pivotal role in tumor cells. Unlike COX‐1 that is expressed constitutively in most cells, COX‐2 is produced in response to growth factors and cytokines 149, 150. Once synthesized, prostaglandin‐2 (PGE‐2) acts in an autocrine and paracrine manner through four receptors to direct epithelial–mesenchymal transition, angiogenesis, HIF‐1 transcription, acid oxidation production, chemo‐resistance, M2 polarization, and Treg and MDSC recruitment. Furthermore, a crosstalk between the immunosuppressive microenvironment and the EGFR pathway activates several signal transduction cascades, including the MAPK, AKT, and PI3K pathways, and subsequent tumor growth and immunosuppression 146.

Preclinical studies found that COX inhibition could enhance the efficacy of anti‐PD‐1 blockade 151, 152. Zelenay and colleagues inoculated Ptgs2‐deficient and BRAFV600E mutated cells in WT mice and found that the loss of COX‐2 expression led to a significant decrease in immunosuppressive cytokine (IL‐6) and chemokine (CXCL1) expression and a simultaneous marked increase in immune‐stimulating factors (IFN‐g, T‐bet, CXCL10, IL‐12, and IFN‐I) and costimulatory molecules 153. Unlike in COX‐deficient tumors, DCs were absented in COX‐ competent tumors. More interestingly, in the same study, mice were randomly assigned to receive aspirin, celecoxib, or anti‐PD‐1 in monotherapy or the combination of a COX inhibitor plus anti‐PD‐1. As expected, the combination promoted a much more rapid tumor regression, with the eradication of BRAFV600E melanoma cells. This study suggests that the association of COX inhibitors and immune checkpoint blockers could enhance the efficacy of immunotherapy and prevent resistance development.

#### Photodynamic therapy in combination with CTLA‐4 blockade

Recent studies have shown that photodynamic therapy (PDT) treatment has the ability to activate the tumor‐specific immune responses by producing tumor‐associated antigens from tumor cell residues, which afterward may be processed by APCs such as DCs and then presented to T cells 154, 155. It is known that the immunological memory response, which is the hallmark feature of adaptive immunities, plays crucial roles in protecting organisms from the second attack of pathogens including tumor cells 156. That is to say, upon a second encounter with the same pathogens, memory T cells can rapidly respond and mount faster and stronger immune responses than the first time the immune system response 157. It is generally recognized that the underlying mechanisms of the combination therapy with ideal inhibition activities on the growth of both primary and distant tumors, as well as the immune memory protection to prevent tumor relapse, may be explained as follows.

The PDT destruction of primary tumors would generate a pool of tumor‐associated antigens to trigger specific immune responses, which were then amplified by UCNP‐Ce6‐R837‐based PDT as the immune adjuvant, which combined with T‐lymphocyte‐associated protein 4 (CTLA‐4) blockade would effectively induce the generation of TEM‐based immune memory response to prevent tumor relapse, similar to the functions of cancer vaccines. More significantly, PDT with UCNP‐Ce6‐R837 in combination with the CTLA‐4checkpoint blockade not only showed excellent efficacy in eliminating tumors exposed to the NIR laser but also resulted in strong antitumor immunities to inhibit the growth of distant tumors left behind after PDT treatment. Furthermore, such a cancer immunotherapy strategy has a long‐term immune memory function to protect treated mice from tumor cell challenge 158. This work presents an immune‐stimulating UCNP‐based PDT strategy in combination with CTLA‐4 checkpoint blockade to effectively destroy primary tumors under light exposure, inhibit distant tumors that can hardly be reached by light, and prevent tumor reoccurrence via the immune memory effect.

In summary, this work has demonstrated the great potency of integrating UCNP‐based PDT with cancer immunotherapy to realize a remarkable synergistic therapeutic outcome in eliminating primary tumors, inhibiting distant tumors, and preventing tumor relapse. While immunotherapy has become a highly promising paradigm for cancer treatment in recent years, it has long been recognized that PDT has the ability to trigger antitumor immune responses. However, conventional PDT triggered by visible light has limited penetration depth, and its generated immune responses may not be robust enough to eliminate tumors.

#### Regulation of the Foxp3 expression in tregs by curcumin

Curcumin is the main active ingredient of the golden spice *Curcuma longa*. More and more evidences suggest that curcumin may be an effective chemical reagent, and its targets are the various molecular signaling pathways involved in the carcinogenesis 159. Recent studies have demonstrated that curcumin repressed the expression of Foxp3 in Tregs 160. As Foxp3 bound T‐bet, the IFN transcription factor, to form a complex, to prevent the IFN‐*γ* expression in CD4+ T cells 161, the inhibition of Foxp3 by curcumin resulted in the IFN‐*γ* expression in the CD4+ T cells. As Th1 cells are one of the important antitumor effector cells 162, these results have expanded our knowledge in the understanding of the antitumor effect of curcumin, which is capable of regulating the property of Tregs in patients with CRC. It has been reported that Tregs are an important cell populationin the tumor tolerance, and inhibition of Tregs is one of the approaches to break down the tumor tolerance 163, 164. Thus, the administration with curcumin may contribute to regulating tumor tolerance.

It has been proved that another in‐depth mechanism by which the curcumin therapy suppressed the levels of Foxp3 in Tregs 165. The suppression of Foxp3 resulted in the increase in T‐bet levels in the Tregs leading the Tregs to be converted to IFN‐*γ*‐producing Th1 cells. Lee et al. have found that such a conversion was mediated by dendritic cell‐derived molecules, which interacted with the Toll‐like receptors on Tregs 166. As curcumin suppressed the expression of Foxp3, the T‐bet was liberated, the IFN‐*γ* was increased in the cells, and the nuclear translocation of p65 and c‐Rel was markedly decreased, which was critical for Foxp3 and CD25 expression after curcumin stimulation 167.

#### Additional combinations with immunotherapy

A large number of studies have been conducted on drugs that may block immune factors, such as LAG‐3 or indoleamine 2,3‐dioxygenase (IDO), which were studied in phase I trials, combined with PD‐L1 or PD‐1inhibitors 168. Interestingly, the decreased recurrence risk and improved survival of CRC is associated with tryptophan (Trp) concentration, which increased the constitutive IDO expression for evading response of cellular immune response 169.

Drugs that act as direct immune stimulators, such as 4‐1BB (CD137) and KIR, have also been studied in a variety of combinations, including PD‐1inhibitor. Anti‐PD1 and anti‐CD137 mAb act on T cells that express these receptors on their plasma membrane presumably as a consequence of an antigencognate activation process 170. Hence, the main mechanism of action is exerted on tumor infiltrating lymphocytes that express such receptors on their surface, thus becoming amenable to pharmacological modulation with the corresponding mAb. In preclinical mouse models, anti‐CD137 and anti‐PD1 mAbs exerted powerful synergistic effects.

There are multiple immunomodulatory compounds and additional checkpoint in the development and phase I survey 171. Cetuximab, for example, is a monoclonal antibody that binds to EGFR and is approved for RAS wild‐type (WT) CRC 172, 173. In clinical studies, cetuximab has been shown to induce EGFR‐specific T‐cell responses and induce antigen diffusion in CRC 174. In patients with metastatic colorectal cancer who received multiple chemotherapy treatments, especially, patients receiving anti‐EGFR treatment showed the strongest neoplasia T‐cell infiltration 175. Both lines of evidence suggested that cetuximab might have an immune‐enhancing effect and may favorably alter the tumor immune microenvironment. Cetuximab immune mechanism may be related therefore to improve the curative effect of combination therapy, treatment, chemotherapy, and immunotherapy of peptides, and ongoing phase Ib/II study demonstrates the role of pembrolizumab and cetuximab in metastatic colorectal cancer.

Altogether, alternative immunotherapeutic approaches have been explored to how to enhance immunotherapy, increase the effector response, and reduce the inflammatory component, such as TGF‐*β* and COX‐2 which are both affected on tumor cells and immune cells in CRC via blockade of related immunosuppressive signaling pathways. In addition, the novel combined approaches, such as immune‐chemotherapy or combo immunotherapy, that could be more effective than chemotherapy or immunotherapy alone (Table 1). Furthermore, it should make clear the extent chemotherapies and targeted agents affect on the immune‐evasive microenvironment, in which Tregs are an important cell populationin the tumor tolerance; therefore, how to discover novel agents to regulate the property of Tregs in patients with CRC is one of the approaches to break down the tumor tolerance.

**Table 1:** Novel combined approaches to enhance immunotherapy

Strategies	The various agents and their targets	Regulated mechanisms	References
Combination of small molecules and checkpoint inhibitors	Anti‐VEGFA with anti‐PD‐1 treatment	VEGF‐A blockade could help sensitize T cells to anti‐PD‐1 treatment and that high VEGF‐A levels may be involved in resistance to this treatment in a mouse model of colorectal cancer.	172
	MEK inhibitor with anti‐PDL1 agent	MEK inhibition induced intratumoral T‐cell accumulation and major histocompatibility complex (MHC) class I upregulation in mouse models, and synergized with immune checkpoint inhibition to promote durable tumor regression. A preliminary clinical trial assessing the combination of a MEK inhibitor with anti‐PDL1 agent showed early signs of efficacy in patients with MSS non‐hypermutated CRC.	173, 174
	Immune modulators and anti‐EGFR therapy	Combination of immune modulators and anti‐EGFR therapy in a RAS wild‐type population, resulting the immune system substantially contributes to the therapeutic effects of mAbs, could produce a synergistic effect in CRC.	175, 176
Combination of COX inhibitors and checkpoint inhibitors	Celecoxib and anti‐PD‐1 monotherapy	COX inhibition could enhance the efficacy of anti‐PD‐1 blockade that the loss of COX‐2 expression leads to a significant decrease in immunosuppressive cytokine (IL‐6) and chemokine (CXCL1) expression and a simultaneous marked increase in immune‐stimulating factors (IFN‐g, T‐bet, CXCL10, IL‐12 and IFN‐I) and costimulatory molecules. This suggests that the association of COX inhibitors and immune checkpoint blockers could enhance the efficacy of immunotherapy and prevent resistance development.	1, 179, 180
Photodynamic Therapy in Combination with CTLA‐4 blockade	UCNP‐Ce6‐R837‐based PDT combined with CTLA‐4 blockade	PDT treatment has the ability to activate the tumor‐specific immune responses by producing tumor‐associated antigens from tumor cell residues, which afterward may be processed by APCs such as DCs and then presented to T cells. PDT combined with CTLA‐4 blockade would effectively induce the generation of TEM‐based immune memory response to prevent tumor relapse.	181, 182
Regulation of the Foxp3 expression in Tregs	Curcumin could represse the expression of Foxp3 in Tregs	As Foxp3 bound T‐bet, the IFN transcription factor, to form a complex, to prevent the IFN‐*γ* expression in CD4+ T cells, the inhibition of Foxp3 by curcumin resulted in the expression of IFN‐*γ* in the CD4+ T cells. As curcumin suppressed the expression of Foxp3, the T‐bet was liberated, the IFN‐*γ* was increased in the cells, the nuclear translocation of p65 and c‐Rel was markedly decreased, which is critical for Foxp3 and CD25 expression after curcumin stimulation.	187, 188, 189
Additional Combinations with Immunotherapy	Block suppressive immune factors, such as indoleamine 2,3‐dioxygenase (IDO) or LAG‐3, combined with PD‐1 or PD‐L1 inhibitors	The IDO1 is a heme enzyme that catabolizes tryptophan (Trp) into kynurenine, while IDO catalyzes oxidative catabolism of tryptophan. The Trp metabolite production and Trp depletion in TME lead to inhibit T‐cell responses, including increased T‐cell apoptosis, naive T cells differentiation into T regulatory cells, and reduced T‐cell proliferation. Consequently, tumor‐specific T‐cell response could be inhibited by IDO expression, and IDO inhibition can improve T‐cell therapy for cancers.	195, 196
	Drugs that are capable of acting as direct immune stimulators, such as KIR and 4‐1BB (CD137), combinations with PD‐1 inhibition	Anti‐PD1 and anti‐CD137 mAb act on T cells that express these receptors on their plasma membrane presumably as a consequence of an antigencognate activation process. Hence, the main mechanism of action is exerted on tumor infiltrating lymphocytes that express such receptors on their surface, thus becoming amenable to pharmacological modulation with the corresponding mAb. In preclinical mouse models, anti‐CD137 and anti‐PD1 mAbs exert powerful synergistic effects.	123
	Cetuximab is a monoclonal antibody and binds to the epidermal growth factor receptor (EGFR)	Cetuximab has been demonstrated to induce an EGFR‐specific T‐cell response as well as induce antigen spreading in CRC. Cetuximab might have an immune‐enhancing effect and may favorably alter the tumor immune microenvironment.	[184‐186]

### Novel strategy to combine with Chemotherapy

#### TNF treatment in combination with melphalan

Single injection of tumor vascular targeting of immune agents such as immune‐cytokine tumor necrosis factor (TNF) coupled with chemotherapeutic melphalan has been shown synergistic therapeutic effects toward different types of tumors 176. Preclinical studies using WEHI‐164 and C51 tumor mouse models showed that antitumor T‐cell immune‐specific responses are induced and correlated with protection and memory, resulting in a “therapy‐induced antitumor vaccination” 177, 178. However, each type of murine tumor studied responds in a different manner in terms of tumor rejection, which could indicate involvement of additional mechanisms employed by the tumor cells to evade T‐cell effector arms or, alternatively, that optimal cell immune stimulation was not still reached.

The crosstalk between NK and DCs might be a crucial point in the regulation of the whole immune defense against tumors and viruses 1, 179, 180, 181. Accumulating evidences have indicated that the cytokine‐producing Th cell capacity, Th cell polarization, as well as migration and stimulatory functions of DCs, could be regulated by activated NK cells 182, 183. On the other hand, the effector functions of NK cells could depend on stimulatory interactions with mature DCs 184, 185. Study has reported that L19mTNF treatment in combination with melphalan in the WEHI‐164 tumor model reduced Treg cells and induced a long‐lasting T‐cell‐mediated immune response involving CD4+ and CD8+ T cells 177, 178. As NK cells and DCs could be involved very early in the immune response, TNF from NK cells could play a relevant role in the maintaining the activation status of DCs. Moreover, a significant increase in the percentages of CD4+ and CD8+ T cells associated with an increase in functional cytotoxic NK cells was found in the spleens of WEHI‐164‐treated mice. These results highlighted the active role played by NK cells in modulating DC maturation, enhancing APC capacity and triggering T‐cell activation following combined therapy, as it has been already demonstrated in other tumor cell systems 179, 180. The present work on L19mTNF/melphalan therapy added new insights in the antitumor functions of NK cells and DCs in the early phases of the tumor response to therapy.

Taken together, these data indicate that NK/DC crosstalk stimulates DCs to promote Th cell proliferation and maturation, which in turn “license” cytotoxic T cells, which are the final effectors. This NK‐DC‐Th‐Tc mechanism could also be functional when L19mTNF is used in combination with IL‐2 186, or with gemcitabine 187, and could provide clues for sensitizing resistant tumors to immune checkpoint blockade therapy 188.

#### The blockade of VEGF signaling in combinations with chemotherapy

One of the main choice treatments for colorectal cancer is chemotherapy using multiple anticancer drugs, such as irinotecan, oxaliplatin, and 5‐fluorouracil (5FU) 189, 190. Chemotherapy resistance has been attributed to many reasons including dysfunctional membrane transport, resistance to autophagy, apoptosis, epigenetic changes, and persistence of stem cell‐like tumor cells 191, 192. Classical cytotoxic therapies are thought to have an impact on tumor microenvironment. It is believed that therapeutic induced cell death can be immunogenicity and promote expression of tumor antigen, which may cause adaptive immune response. Chemotherapy and radiotherapy are all interpreted as having such properties, although the optimal program for immunogenic cell death induction remains to be determined, and the true size of this effect remains unclear.

It has been observed that both oxaliplatin and 5‐FU are considered to have a good effect 193. Based on this principle, FOLFOX combined the use of pUNK lizumab in two studies for gastric cancer or colon cancer. The combination of FOLFOX and bevacizumab can reduce the MDSCs of granulocytes, increase the frequency of pro‐inflammatory helper t cells (Th17), and provide a good microenvironment for the treatment of immunocheckpoint inhibitors 194. The comprehensive effect of chemotherapy and anti‐angiogenic factors on immune checkpoint therapy is being evaluated. The combination of FOLFOX, bevacizumab and atezolizumab was studied in the cohort of 30 patients. Of the population, 11 (48%) showed a partial response of 48%, with 20/23 (87%) responding to or stabilizing the disease. Tumor biopsy and peripheral blood showed immunological activation 195. It is worth noting that bevacizumab associated with these two VEGF blockade of signal increased CD163+ dendritic cells trafficking, and CD8+ t cells across the trafficking of tumor blood vessels, and not just by ipiliumumab 196.

Collectively, selective and effective regulating antitumor T‐cell immune‐specific responses for anticancer therapy are essential to inhibit CRC cells, and TNF is used as an immune‐cytokine tumor necrosis factor in combination with chemotherapeutic agents, and could provide clues for sensitizing resistant tumors to immune checkpoint blockade therapy. Moreover, the novel stratergy to combine effects of chemotherapy and anti‐angiogenic agents on immune checkpoint therapy by provoking an adaptive immune response have been listed (Table 2). Future research should focus on the possibility of bioactive agents to regulate NK/DC crosstalk that stimulates T cells activity and DCs activation. However, it is still not clear whether the results represented a separate chemotherapy case for the group. Because there is no difference in possible response speed, persistence can be improved, and mature data, including PFS and operating systems, will provide information.

**Table 2:** Novel strategy to combinate with chemotherapy

Strategies	The various drugs and their targets	Regulated Mechanisms	References
Combination of tumor necrosis factor (TNF) treatment	L19mTNF treatment in combination with melphalan	L19mTNF treatment in combination with melphalan in the WEHI‐164 tumor model reduced Treg cells and induced a long‐lasting T‐cell‐mediated immune response involving CD4+ and CD8+ T cells. As NK cells and DCs could be involved very early in the immune response, TNF from NK cells could play a relevant role in the maintaining the activation status of DCs.	[150,151]
	L19mTNF in combination with IL‐2, or with gemcitabine	NK/DC crosstalk stimulates DCs to promote Th cell proliferation and maturation, which in turn “license” cytotoxic T cells (Tc), which are the final effectors. This NK‐DC‐Th‐Tc mechanism could also be functional when L19mTNF is used in combination with IL‐2, or with gemcitabine, and could provide clues for sensitizing resistant tumors to immune checkpoint blockade therapy	[191‐193]
The blockade of VEGF signaling in Combinations with Chemotherapy	FOLFOX is being combined with bevacizumab	FOLFOX and bevacizumab may decrease granulocytic MDSCs and increase pro‐inflammatory helper T‐cell (Th17) frequency, rendering a favorable microenvironment for immune checkpoint inhibitor treatment	[179]
	The combination of FOLFOX, bevacizumab and atezolizumab	The combination of FOLFOX, bevacizumab and atezolizumab demonstrated partial response 48% with 20/23 (87%) achieving response or stable disease. Tumor biopsies and peripheral blood demonstrate immune activation	[180]
	Bevacizumab combined with ipilimumab	Bevacizumab combined with ipilimumab increased CD163+ dendritic cell trafficking and and CD8+ T‐cell trafficking across the tumor vasculature beyond what was achieved via ipiliumumab alone	[178]

## Discussion

The recently developed immunotherapeutic strategies have yielded remarkable clinical results in many types of tumors including CRC, indicating that indeed a patient's immune system can mount an immune response, which is effective in controlling tumor growth 123. However, a high proportion of patients is resistant or acquires resistance to these therapeutic strategies. In recent years, emerging novel immunotherapeutic approaches could change the CRC landscape. Moreover, selection criteria are necessary to identify patients who may benefit from immune checkpoint inhibitors. To this end, the presence of TILs is one of the most important predictors.

Specifically, DNA MMR and MSI status is now clinically significant to determining whether patients may be eligible for immunotherapy in clinical trials, but the potential predictive factors in MSS patients have been ignored. To date, the predictive role of the differential expression of PD‐1 and PD‐L1 has not been completely clarified, although some evidence suggests that high expression correlates with a better immunotherapy efficacy. As far as we know, only MSI‐H CRC tumors respond to checkpoint inhibition. In this review, the basis for this has been explained and the results are obtained thus far. This apparent limitation could be seen as a point of strength. Although the only available data have come from phase II trials, as phase III trials are currently ongoing, the results achieved so far are exciting 197. Based on these results, the FDA has granted Breakthrough Therapy Designation to pembrolizumab for the treatment of MSI‐H CRC. This paves the way for new therapeutic possibilities but also raises new doubts and questions, some of which concern very practical matters.

Currently, most immunotherapies are still in early‐phase clinical testing for CRC, but their successful use in other types of cancers suggests that they may ultimately prove useful for CRC as well. As the field of immunotherapy continues to evolve, a more comprehensive understanding of drug resistance mechanisms will be mandatory, leading to the development of new strategies to overcome major and acquired resistance to anti‐PD/PDL‐1 antibodies. The inability of the immunotherapeutic strategies used to eradicate cancer‐initiating cells (CICs) that escapeimmune recognition and destruction may give rise to new tumors in the same organ site or through the metastatic colonization in other anatomic sites. Accordingly, identification of novel therapeutic approaches that can eradicate CICs is a major challenge in the CRC therapy area. In consequence, an improved understanding of the interactions of CICs with immune system and with tumor microenvironment may contribute to optimize the available therapies and to design novel combination treatments for CRC therapy.

Importantly, rational combinations of targeted therapies will be required to achieve meaningful effects in different subtypes, with overlapping toxic effects that may further complicate the biomarker–drug codevelopment path. In the setting of actionable genomic alterations detected in tumors samples or ctDNA, for example, an additional layer of complexity is the rarity of most events (such as ERBB2 and MEK1 mutations) and the need to adapt therapies accordingly upon progression. To improve clinical benefit further, it is crucial to understand how residual disease is sustained and how it can be therapeutically tackled. The potential impact of immunotherapy on the cells of resistant clones and the lack of target gene modification is being studied extensively. These combinations include small‐molecule, inhibitors of immunosuppression immunomodulators, and T‐cell costimulatory agents, chemokines, vaccination, targeted agents, cytotoxic drugs, and radiation therapy.

The regulatory effector T cells have been developed using CRC in treatment and will be more advanced in overcoming immune avoidance mechanisms and survival in the tumor immune‐suppressive environment. In addition to technical challenges, there is also lack of understanding of the specific steps to promote tumor development, tumor malignancy, and its eventual transfer to resistance. There are a number of mechanisms that provide metabolic pathways; however, whether these pathways may be the upstream signal transduction mechanism is not yet understood. Therefore, the future work should not be focused on the casual switch to the T‐cell response, but try to discover the regulatory reaction of the T cells based on the necessary immune mechanisms. This immunotherapeutic strategy when developed could exhibit effective immune response and minimize the undesirable effects. Additionally, future efforts should focus on specific tumor antigen recognition by employing the method of highly personalized, as well as developing efficient lymphodepleting scheme before the T‐cell transfers. This could be effective in combination with other treatments, such as molecular agents against relying on oncogenes of tumor and strong regulators in host immune.

## Conflict of Interest

The authors report no conflicts of interests in this work.
